# N^6^-methyladenosine (m^6^A) RNA modification in tumor immunity

**DOI:** 10.20892/j.issn.2095-3941.2021.0534

**Published:** 2022-03-08

**Authors:** Siyi Zheng, Hui Han, Shuibin Lin

**Affiliations:** 1 Center for Translational Medicine, Precision Medicine Institute, The First Affiliated Hospital, Sun Yat-sen University, Guangzhou 510080, China; 2 Department of Otolaryngology, Center for Translational Medicine, Precision Medicine Institute, The First Affiliated Hospital, Sun Yat-sen University, Guangzhou 510080, China

**Keywords:** N^6^-methyladenosine (m^6^A) modification, immune evasion, tumor microenvironment (TME), tumor immunology, immune cells

## Abstract

Growing evidence supports that cancer progression is closely associated with the tumor microenvironment and immune evasion. Importantly, recent studies have revealed the crucial roles of epigenetic regulators in shaping the tumor microenvironment and restoring immune recognition. N^6^-methyladenosine (m^6^A) modification, the most prevalent epigenetic modification of mammalian mRNAs, has essential functions in regulating the processing and metabolism of its targeted RNAs, and therefore affects various biological processes including tumorigenesis and progression. Recent studies have demonstrated the critical functions and molecular mechanisms underlying abnormal m^6^A modification in the regulation of tumor immunity. In this review, we summarize recent research progress in the potential roles of m^6^A modification in tumor immunoregulation, with a special focus on the anti-tumor processes of immune cells and involvement in immune-associated molecules and pathways. Furthermore, we review current knowledge regarding the close correlation between m^6^A-related risk signatures and the tumor immune microenvironment landscape, and we discuss the prognostic value and therapeutic efficacy of m^6^A regulators in a variety of cancer types.

## Introduction

Epigenetics, defined as heritable and potentially reversible changes in gene expression that occur without alterations in the DNA sequence, has received increasing attention in the past few decades^1^. Epigenetic drugs, such as DNA methyltransferase inhibitors and histone deacetylase inhibitors, have been used to restore aberrant levels of epigenetic modifications and have been applied in the clinical treatment of a variety of tumors^2^. Analogously to DNA and histone modifications, RNA modifications have been shown to regulate gene expression at the post-transcriptional level and have become a major research focus in recent years^3^. More than 100 post-transcriptional modifications have been confirmed to occur in cellular RNAs. Among them, N^6^-methyladenosine (m^6^A), first identified in the 1970s, is the most abundant modification in most eukaryotic mRNAs^4^. The rapid development of RNA immunoprecipitation-sequencing methods has uncovered the transcriptome-wide m^6^A modification landscape. The deposition of the m^6^A modification is not random but occurs mainly in RRACH sequences (where R = A or G, and H = A, C, or U) and is concentrated near the stop codon, 3′UTR, and long internal exons^5^. The m^6^A modification regulates a variety of biological functions, including tissue development^6^, circadian rhythms^7^, the DNA damage response^8^, and sex determination^9^. Accumulating studies indicate that aberrant m^6^A methylation levels contribute to tumorigenesis and cancer progression. Therefore, targeting dysregulated m^6^A regulators, similarly to other epigenetic regulators, may provide an attractive potential strategy for cancer therapy.

The interaction between cancer and the immune system is complicated. During tumor progression, cancer cells can evade host immune surveillance and weaken the immune response through various mechanisms, including decreasing the expression of tumor-associated antigens or promoting the formation of an immune-inflammatory microenvironment. Although cancer immunotherapy has been shown to be effective across a broad range of cancer types, only a subset of patients with cancer show clinical responses, because immune checkpoints can be dysregulated or hijacked as a mechanism of immune resistance. Therefore, revealing novel molecular mechanisms on tumor immune resistance can improve the efficacy of tumor immunotherapy.

In recent years, epigenetic drugs such as inhibitors of DNA methyltransferases and histone deacetylases have been demonstrated to effectively reverse immune suppression. Those epigenetic inhibitors function *via* several mechanisms, such as enhancing the expression of tumor-associated antigens, components of the antigen processing and presenting machinery pathways, immune checkpoint inhibitors, chemokines, and other immune-associated genes^2^.

Recent studies have revealed the correlation between m^6^A methylation and tumor immunity, thus broadening the understanding of m^6^A methylation and tumor immune regulation, and improving the efficacy of immune checkpoint blockade therapy and overcoming immune resistance.

In this review, we focus on the interplay among m^6^A modification, functional maintenance of the immune system, and the landscape of the tumor immune microenvironment, to provide a theoretical foundation for understanding the crosstalk between m^6^A modification and anti-tumor immunity. In addition, we highlight the promise of combining targeting of m^6^A regulators and immunotherapy to restore immune recognition and immunogenicity for the effective treatment of cancers.

### Composition of the RNA m^6^A methylase complex

**Figure 1:**
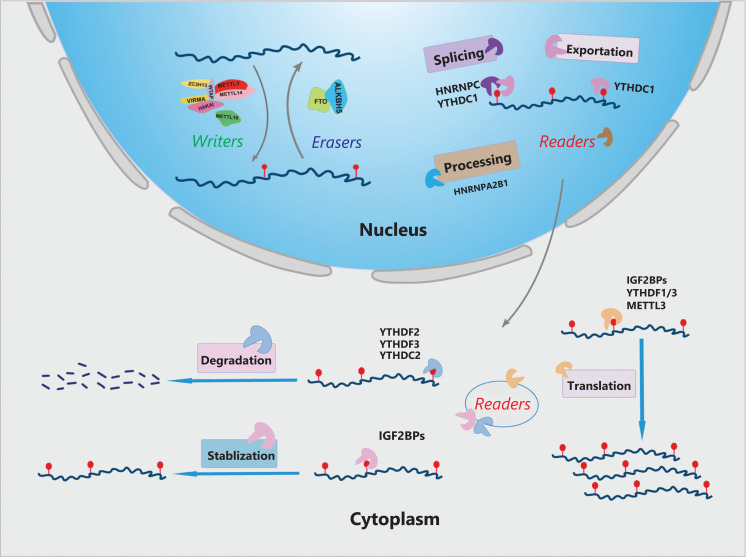
Overview of RNA N^6^-methyladenosine (m^6^A) modification. The m^6^A modification is modulated by m^6^A “writers”, “erasers”, and “readers”. m^6^A “writers” are methylase complexes including METTL3, METTL14, ZC3H13, RBM15, VIRMA, HAKAI, WTAP, and METTL16. “Erasers” are demethylases (FTO, ALKHB5) that remove methyl groups. The m^6^A-containing RNAs are recognized by “readers”, which are involved in multiple processes of RNA metabolism, such as primary miRNA processing, alternative splicing, mRNA stabilization, translation, and degradation.

Beyond the epigenetic modification of DNA and histones, RNA modification has gradually become a major research focus. More than 170 RNA chemical modifications have been identified^10^. m^6^A is the most prevalent and reversible internal modification in mammalian messenger and noncoding RNAs. Emerging evidence indicates that abnormal m^6^A levels are strongly associated with tumor initiation and progression in various types of cancers. m^6^A modification is dynamically regulated by methyltransferases (“writers”) and demethylases (“erasers”), and is recognized by various readers, functions attributed to m^6^A cover nearly all aspects of mRNA processing, ranging from pre-mRNA splicing, export, translation and stability (**Figure 1**)^11^.

Notably, “writers” are composed of a multicomponent methyltransferase complex that deposits m^6^A modifications. Methyltransferase-like 3 (METTL3), Methyltransferase-like 14 (METTL14), and Wilms tumor1-associated protein (WTAP) were initially identified in this complex^12,13^. Among them, METTL3 is the key methylase that catalyzes methylation, by transferring a methyl from S-adenosine methionine (SAM) to the adenine moiety of the target. METTL14 forms a heterodimer with METTL3 and stabilizes the METTL3-RNA interaction by providing a platform for RNA binding^12,13^. METTL3 has been found to promote translation of certain oncogenes in human lung cancer^14^. Interestingly, METTL3 recruits the translation initiation complex to target mRNAs through direct interaction with eIF3b. The METTL3-eIF3h interaction is required for enhanced translation, the formation of densely packed polyribosomes, and oncogenic transformation^15^. Similarly, WTAP assists in the localization of heterodimers to nuclear plaques, thus facilitating the recruitment of the complex to specific RNA targets^16^. Apart from these 3 core components, VIRMA/HAKAI/ZC3H13 have also been identified to be part of the entire methyltransferase complex^17^. Unlike other methyltransferase complexes, METTL16 is a newly discovered m^6^A “writer”, which binds a range of non-coding RNAs^18^ and interacts with pre-mRNA, thus regulating the splicing process^19^.

The removal of the m^6^A modification is performed by demethylases termed m^6^A “erasers”. The first demethylase, fat mass and obesity-associated protein (FTO), was discovered in mammals in 2008^20^. To date, only 2 m^6^A demethylation enzymes have been found in eukaryotes. AlkB homologue 5 (ALKBH5), the second identified “eraser”, has been implicated in the reversible removal of methyl groups from the m^6^A modification specifically. The discovery of “erasers” has brought another dimension to the study of RNA epigenomics. Mechanistically, the unique C-terminal long loop domain in FTO enables it to demethylate the methylated single-stranded DNA or RNA. FTO specifically removes m^6^A marks from the GGACU and RRACU motifs *via* oxidative demethylation activity. Moreover, demethylation mediated by ALKBH5 is critical for RNA splicing^21^. The dynamic regulation of m^6^A by methyltransferases and demethylases provides an additional layer of gene expression regulation at the post-transcriptional level.

Various m^6^A “readers” specifically recognize the m^6^A residues and subsequently exert biological functions in RNA metabolism and multiple biological processes. The m^6^A “readers” are divided into several protein families: YT521-B homology (YTH) domain family, insulin-like growth factor 2 mRNA-binding proteins (IGF2BPs), and heterogeneous nuclear ribonucleoproteins (HNRNPs). YTHDF2, the first recognized “reader”, decreases the stability of targeted transcripts and promotes mRNAs degradation by recruiting the CCR4-NOT deadenylase complex^22^. Subsequently, more YTH domain proteins were discovered, including YTH domain family proteins (YTHDF1 and YTHDF3), and YTH domain-containing proteins (YTHDC1 and YTHDC2)^23–26^. Further studies have indicated that YTHDF3 promotes the translation and degradation of m^6^A transcripts *via* different mechanisms, such as by regulating its own mRNA translation and increasing its protein expression^27^, as well as interacting with ribosomes and participating in the translation initiation and elongation^28^. In addition, YTHDC2 has been revealed to regulate the stability of m^6^A-targeted mRNA and recruit the RNA degradation machinery. Knockdown of YTHDC2 has been demonstrated to increase m^6^A-modified transcripts^29^. Moreover, YTHDC1 contributes to RNA splicing and export^26^. By promoting exon inclusion in targeted mRNAs through recruiting pre-mRNA splicing factor SRSF3 (SRp20) while blocking SRSF10 (SRp38) mRNA binding, YTHDC1 regulates mRNA splicing and consequently mediates the transfer of “targeted mRNAs” from the nucleus to the cytoplasm^30^.

Huang et al.^31^ have provided compelling evidence that, in contrast to the mRNA-decay-promoting function of YTHDF2, IGF2BPs—a distinct family of m^6^A “readers” that preferentially recognize thousands of m^6^A-modified mRNA transcripts through the consensus GG(m^6^A)C sequence—promotes mRNA stability and translation in an m^6^A-dependent manner, thus globally affecting gene expression output. Moreover, m^6^A modification changes the secondary structure of RNA, promotes the binding of HNRNPC and HNRNPA2B1 and other nuclear heterogeneous ribosomal protein family readers, and participates in primary microRNA processing and alternative splicing^32^.

In short, “writers” and “erasers” determine the m^6^A levels in specific transcripts; these modifications are interpreted by “readers” and have roles in multiple biological functions and diverse processes in RNA fate, including primary miRNA processing, alternative splicing, and mRNA stabilization, translation, and degradation.

### m^6^A modification involved in immune cells

In the past several decades, m^6^A methylation has been widely demonstrated to play complex roles in the homeostasis of the immune system, such as the recognition of, and response to, invading pathogens, and exogenous or aberrant endogenous RNAs^33–35^. Several investigations have demonstrated the crosstalk between m^6^A regulators and their regulation in general biology in immune cells. Indeed, studies increasingly indicate that RNA m^6^A methylation is associated with tumor-intrinsic oncogenic pathways. In this section, we review the recent progress in understanding of the roles of m^6^A modification regulation, with a focus on the anti-tumor processes of both innate and acquired immune cells, including macrophages, dendritic cells, lymphocytes, and natural killer cells.

### m^6^A modification in macrophages

Macrophages are crucial mediators of tissue homeostasis that have phagocytic, antigen presentation, and cytokine secretion functions. Under different tissue microenvironments and cytokines, macrophages can polarize into 2 phenotypes: macrophage type 1 (M1) and macrophage type 2 (M2)^36,37^. In the tumor microenvironment (TME), M2 has pro-tumorigenic properties^38^, whereas M1 is involved in promoting anti-tumor immunity^39^. Recent studies have shown that the m^6^A modification is involved in regulating the polarization of macrophages. STAT1 is the main transcription factor regulating macrophage polarization into M1. METTL3 increases the transcriptional stability and expression of STAT1 mRNA in an m^6^A-dependent manner, thus promoting the polarization of macrophages^40^.

Intriguingly, recent research has suggested that METTL3 is required for macrophage-mediated innate immunity. Mechanistically, METTL3 deficiency significantly decreases m^6^A modification of IRAKM mRNA, thus diminishing its degradation, resulting in higher IRAKM levels, and suppressing macrophage activation *via* the TLR4 signaling pathway^41^.

Similarly, a recent study has revealed a non-cell-intrinsic, tumor-suppressing function of METTL3: ablation of METTL3 in macrophages has been found to contribute to the formation of an immunosuppressive microenvironment, including increased regulatory T cell (Treg) infiltration, and fewer Th1 cells and IFN-γ^+^CD8^+^ cells, thus facilitating tumor growth and metastasis. Mechanistically, METTL3 depletion impairs the YTHDF1-mediated translation of SPRED2, thereby enhancing the activation of NF-κB and STAT3 through the ERK pathway. Moreover, loss of METTL 3 impairs PD-1 blockade therapeutic efficacy in B16 tumors^42^. Beyond METTL3 loss, a deficiency in Mettl14 or Ythdf2 in macrophages promotes the accumulation of EBI3 mRNA in an m^6^A-dependent manner, thereby triggering CD8^+^ T cell dysfunction, preventing CD8^+^ T cell infiltration, and accelerating tumor progression^43^.

Overall, these studies illustrate that m^6^A modification in macrophages might serve as a promising target for tumor immunotherapy.

### m^6^A modification in dendritic cells

Dendritic cells (DCs), another antigen-presenting cell type involved in the immune response, link innate and adaptive immunity. DCs interact with T cells in both tissue lymph nodes and TME, thus promoting functional activation^44^. Dysregulation of DC-mediated immune activation is generally accepted to be closely associated with several pathological conditions. DC vaccines also have been a promising strategy for cancer treatment in recent years. Exploration of the regulatory mechanisms of the m^6^A epi-transcriptome has increased understanding of m^6^A-mediated DC function and supported translational exploitation of m^6^A-based tumor immunotherapy.

Recently, RNA m^6^A modification involved in DCs has been reported to be critical for anti-tumor immunity effects. Loss of YTHDF1 in DCs enhances the cross-presentation of tumor antigens and the cross-activation of CD8^+^ T cells *in vivo*. Mechanistically, YTHDF1 increases the translation of lysosomal protease transcripts with m^6^A modification. In addition, the therapeutic efficacy of PD-L1 checkpoint blockade immunotherapy is greater in *Ythdf1^–/–^* mice than wild-type mice, thus indicating that YTHDF1 is a potential therapeutic target in anti-tumor immunotherapy^45^.

### m^6^A modification in lymphocytes

Two types of lymphocytes are critical in specific immune responses: T lymphocytes (T cells) and B lymphocytes (B cells). Research on the involvement of m^6^A modification in lymphocyte development and tumorigenesis remains in early stages. However, studies have focused on the crosstalk between RNA m^6^A methylation and abnormalities in lymphocyte homeostasis. For instance, a study by Li et al.^46^ has demonstrated that deletion of METTL3 in CD4^+^ T cells inhibits activation of the IL-7/STAT5 signaling pathway, which is associated with the differentiation and development of T cells and contributes to imbalanced naïve T cell homeostasis. Moreover, another study has uncovered the effects of m^6^A methylation in Tregs, thus suggesting that METTL3 deficiency in Tregs contributes to elevated expression of Socs genes targeting the IL-2/STAT5 signaling pathway^47^. In addition, YTHDF1 deficiency in DCs intensifies the early steps of T cell priming against tumor neoantigens. Furthermore, the anti-tumor response is completely abrogated in *Ythdf1^–/–^* mice lacking CD8^+^ T cells, thus highlighting the involvement of m^6^A modification in the regulation of CD8^+^ T cells and tumorigenesis. Similarly, RNA m^6^A modification has been demonstrated to control the early development of B cells. More recently, a study has indicated that deletion of METTL14 specifically in B cells leads to severe defects in B cell development in mice, with inhibition of interleukin-7-induced pro-B cell proliferation and blocking of the large-to-small pre-B cell transition^48^. These studies have demonstrated the important regulatory roles of m^6^A modification in T cells or B cells as well as their involvement on tumor progression.

### m^6^A modification in NK cells

Natural killer (NK) cells, the predominant innate lymphoid cells, are critical mediators of host immunity against virus-infected and cancerous cells^49^. In general, NK cells release cytotoxic molecules, which induce apoptosis or pyroptosis. In addition, NK cells enhance the functions of other innate and adaptive immune cells *via* the secretion of several cytokines and chemokines^50^.

A recent report by Ma et al.^51^ has indicated that YTHDF2 positively regulates NK cells’ anti-tumor and antiviral activity. Briefly, YTHDF2 promotes the secretion of perforin, granzyme B, and IFN-γ, and decreases melanoma metastasis. Depletion of YTHDF2 in NK cells significantly impairs NK cell anti-tumor and antiviral immunity *in vivo*. Moreover, YTHDF2 has been demonstrated to maintain the homeostasis and maturation of NK cells. Subsequently, a related study unveiled a critical role of METTL3-mediated m^6^A methylation in the homeostasis and anti-tumor immunity of NK cells. In that study, mice with conditional Mettl3 depletion in NK cells showed aggravated tumor progression and shortened survival, accompanied by suppressed infiltration and impaired tumor immunosurveillance functions of NK cells in the TME^52^. Overall, these observations have elucidated the role of m^6^A modification in the anti-tumor immunity of NK cells, highlighting new biological roles of m^6^A modification in tumor immune regulation in NK cells.

### m^6^A modifications involved in tumor immune evasion

Generally, according to the different characteristics of the TME, tumor immune evasion can be divided into 2 types either with or without a T cell inflamed phenotype. The former type comprises infiltrating T cells and an extensive cytokine profile because of the activation of the immune system. This type resists immune attack *via* immune system suppressive pathways. The latter type lacks infiltrating T cells, exhibiting immune escape by immune system evasion^53^. Tumor cells can create an immune-inflammatory suppression microenvironment to avoid immune surveillance or weaken the immune response. In addition, the TME regulates tumor progression and drug resistance through altering cell-cell and extracellular matrix adhesion and modulating immune responses.

This section focuses on m^6^A modifications involved in the regulation of immune-associated molecules and tumor immune-associated pathways involved in the formation of the tumor immune environment and tumor immune evasion, as well as drug resistance.

### m^6^A regulators involved in the expression of immune inhibitory molecules

Programmed death-ligand 1 (PD-L1) is a primary immune inhibitory molecule expressed on tumor cells that promotes immune evasion. In the past few years, immune checkpoint blockade therapies blocking programmed cell death protein 1 (PD-1) and PD-L1 have shown tremendous benefit in patients with various types of tumors. The PD-1/PD-L1 blocking response is associated with numerous tumor-intrinsic and tumor immune microenvironment characteristics^54,55^; as a result, a considerable proportion of patients show no response or develop resistance to PD-1/PD-L1 blockade therapy. Therefore, further understanding of the molecular mechanism of PD-1 regulation may aid in enhancing the therapeutic efficacy of checkpoint blockade.

Compared with untreated *Ythdf1^−/−^* mice or anti-PD-L1-treated WT mice, *Ythdf1^−/−^*- mice treated with PD-L1 checkpoint blockade immunotherapy have been found to show more complete tumor regression, thus suggesting YTHDF1 as a potential target in anti-tumor immunotherapy. In addition, Wang et al.^56^ have shown that, in colorectal carcinoma and melanoma, depletion of METTL3 and METTL14 enhances the response to anti-PD-1 blockade therapy through stabilizing Stat1 and Irf1 mRNA. These findings have increased awareness of the function of RNA methylation in response to anti-PD-1 treatment, and suggested METTL3/14 and YTHDF1 as potential therapeutic targets in anti-PD-1 checkpoint blockade immunotherapy.

Remarkably, inhibition of FTO in melanoma has been found to suppress tumorigenicity and to increase the m^6^A levels in PD-1, CXCR1, and SOX10, hence enhancing the decay of these m^6^A-targeted mRNAs by YTHDF2. More importantly, *in vivo*, selective blocking of FTO restores the IFN-γ response and increases sensitivity to anti-PD-1 therapy treatment^57^. Similarly, Tsuruta et al.^58^ have revealed that the RNA N^6^-methyladenosine demethylase FTO regulates PD-L1 expression in colon cancer cells. Depletion of FTO decreases PD-L1 expression in an IFN-γ signaling-independent manner. This result has provided new insights into the regulation of PD-L1 expression by RNA modification. A recent study has also indicated that in intrahepatic cholangiocarcinoma, tumor-intrinsic ALKBH5 inhibits the cytotoxicity of T cells by sustaining tumor cell PD-L1 expression. Moreover, ALKBH5 decreases the infiltration of myeloid-derived suppressor-like cells in the tumor immune microenvironment, and the ALKBH5-PD-L1-regulating axis has been confirmed^59^. Together, these studies have identified FTO and ALKBH5 are direct targets of PD-1 or PD-L1, and have revealed a new PD-L1 regulatory mechanism involving mRNA epigenetic modification. More importantly, these findings provide a basis for substantially improving the therapeutic efficacy of checkpoint blockade therapy and inhibiting tumor immune evasion.

Beyond PD-L1, CD47 is a key receptor expressed in tumor cells and is involved in immune escape in the TME. CD47 is a transmembrane glycoprotein well known for its “self/do not eat” signal in normal cells. Mechanically, the interaction between CD47 and macrophage signal regulatory protein alpha (SIRPα) inhibits phagocytosis by macrophages. The level of CD47 has also been reported to correlate with invasion and metastasis in various malignancies including leukemia, lymphoma, multiple myeloma, and several solid tumors, such as breast cancer and hepatocellular carcinoma (HCC)^60^. Most recently, Fan et al.^61^ have revealed the regulation of CD47 expression in HCC. Mechanically, METTL3/IGF2BP1 positively regulate CD47 expression in an m^6^A-dependent manner, and CD47 mediated EMT has been found to contribute to microwave ablation-induced metastasis in HCC. These findings shed new light on the crosstalk between CD47 and m^6^A modification.

### m^6^A modification involved in immune-associated signaling pathways

Apart from the above immune inhibitory molecules, several immune-associated signaling pathways also regulate the tumor immune microenvironment. Here, we summarize the crosstalk between m^6^A modification and key molecules in immune-associated signaling pathways, and discuss the related potential therapeutic strategies.

### HIPPO/YAP signaling pathway

The HIPPO signaling pathway plays a crucial role in priming the appropriate immune responses to viruses, bacteria, and several carcinogenic factors for the maintenance of homeostasis, among which the core regulatory protein Yes-associated protein (YAP) participates in tumor immunoregulation. When YAP is in a low phosphorylation state, it functions as a transcriptional co-activator promoting downstream target gene expression. Activated YAP is beneficial in recruiting tumor-associated macrophages^62^.

In prostate cancer cells and pancreatic ductal cancer cells, high expression of YAP upregulates the secretion of the CXCL5 chemokine, promotes the recruitment of MDSCs, and protects tumor cells from immune clearance^63,64^, whereas phosphorylation and inactivation of YAP lead to a decrease in Treg number and activity, thereby promoting anti-tumor effects^65^. In addition, YTHDF2 orchestrates the epithelial-mesenchymal transition/proliferation dichotomy *via* HIPPO/YAP signaling, thus suggesting that YTHDF2 has potential value as a target for pancreatic cancer^66^. These findings demonstrate that YTHDF2 is involved in tumor immune evasion.

YAP activation is crucial for cancer progression, and m^6^A modified transcripts of lncRNA have been demonstrated to regulate YAP activation in colorectal cancer (CRC). In CRC tissue, YTHDF3 expression is elevated. YTHDF3 recognizes m^6^A-containing GAS5 and accelerates its degradation, thus further activating the YAP signaling pathway in CRC^67^. In addition, Zhang et al.^68^ have uncovered a novel circRNA_104075 related pathway that stimulates YAP in HCC and have provided new evidence that m^6^A modification in the YAP 3´UTR participates in HCC occurrence and development. More recently, studies have shown that m^6^A modification regulates the expression of YAP and may have potential clinical importance in the diagnosis and prognosis of several tumor types such as non-small cell lung cancer (NSCLC). In NSCLC, METTL3 increases the stability and the efficiency of translation of YAP by upregulating YAP m^6^A levels and recruiting YTHDF1/3^69^. Similarly, another study has revealed that, in NSCLC, ALKBH5 modulates YTHDF-mediated YAP expression by removing m^6^A modifications on YAP, and inhibits YAP activity through the miR107/lats2 axis, thus suppressing tumor growth and metastasis^70^. Therefore, effective regulation of YAP m^6^A levels may be a potential therapeutic strategy for NSCLC. Overall, m^6^A modification has been reported to be involved in tumor progression and metastasis through the HIPPO signaling pathway in several studies.

### WNT/β-catenin signaling pathway

Similarly to the HIPPO/YAP signaling pathway, the WNT/β-catenin signaling pathway plays a pivotal role in the maintenance of cellular homeostasis and the functional regulation of several immune cells. It also regulates immunosurveillance mechanisms in the TME under various physiological conditions. Alterations in this pathway are associated with the dysregulation of the anti-tumor immune response. Abnormal activation of the WNT/β-catenin signaling pathway has been observed in a wide range of solid tumors of non-T-cell inflammatory infiltration types, such as bladder cancer^71^, head and neck squamous cell carcinoma^72^, epithelial ovarian cancer^73^, and CRC^74^, beyond metastatic melanoma. In tumor cells with high expression of β-catenin and activation of the WNT pathway, CCL4 secretion is diminished, and the recruitment of DCs into the TME by CCL4 is blocked, thus posing an obstacle to T cell infiltration into the TME^75^. Overall, the WNT signaling pathway is essential for tumor progression and survival, even in the presence of the anti-tumor immune response^76^.

Recently, anomalous expression of m^6^A regulators has been revealed in cancers with abnormal activation of the WNT signaling pathway. For example, in CRC, YTHDF1 recognizes and promotes the translation of methylated FZD9 and WNT6, thus increasing the expression of β-catenin and abnormal activation of the WNT/β-catenin signaling pathway. Knockout of YTHDF1 significantly inhibits the development of CRC^77^. Similarly, METTL3 targets CTNNB1 in hepatoblastoma. Mechanistically, methylation of CTNNB1 transcripts by METTL3 increases the expression of β-catenin, which is involved in the regulation of tumor immunity, thereby increasing the proliferation and tumorigenicity of hepatoblastoma cells^78^. Additionally, in osteosarcoma tissues and cell lines compared with in normal tissue cells, METTL3 is more highly expressed, and it regulates the m^6^A level of LEF1 and activates the WNT/β-catenin signaling pathway, thus playing a critical role in the progression of osteosarcoma^79^. Moreover, in HCC, YTHDF1 promotes the translational output of FZD5 mRNA in an m^6^A-dependent manner and facilitates the progression of HCC, thus acting as an oncogene *via* the WNT signaling pathway^80^.

### Therapeutic and prognostic efficacy of m^6^A regulators

Currently, cancer treatments often include surgery, chemotherapy, radiotherapy, and immunotherapy, although therapy resistance has been demonstrated to be a consequence of multiple factors such as individual variability in drug sensitivity, tumor location, tissue lineage, tumor aggressiveness, and intracellular molecular alteration^81^. The major reason for the failure to eliminate tumors is a lack of comprehensive understanding of the molecular mechanism underlying therapeutic resistance.

The PD-1 blocking response is associated with numerous tumor-intrinsic and tumor immune microenvironment characteristics^54,55^; as a result, a considerable proportion of patients show either no response or drug resistance. Thus, searching for biomarkers predictive of the immunotherapy response and identifying patients who may benefit from treatment might guide more effective immunotherapy strategies and the development of promising therapeutic targets.

Emerging evidence indicates that in multiple cancer types, m^6^A regulators are strongly associated with acquired therapy resistance, including acquired chemoresistance, radioresistance, and resistance to immunotherapy^81^. For instance, in melanoma and CRC, ALKBH5 regulates the anti-PD-1 therapy response by modulating lactate levels and suppressing immune cell infiltration in the TME. Mechanistically, ALKBH5 regulates the expression of Mct4/Slc16a3 and the content of lactate in the TME, thus suppressing the composition of tumor-infiltrating Treg cells as well as MDSCs, and finally regulating the response of PD-1 immunotherapy^82^. In another example, LILRB4 targeted by FTO in AML promotes immune evasion^83^. In melanoma, inhibition of FTO suppresses tumorigenicity and increases the m^6^A levels in PD-1, CXCR1, and SOX10, hence enhancing the decay of these m^6^A-targeted mRNAs by YTHDF2. More importantly, selective blocking of FTO restores the IFN-γ response and sensitizes anti-PD-1 therapy treatment *in vivo*^57^.

Recent studies have consistently demonstrated that the m^6^A-related risk signature (“m^6^A score”), defined by multiple m^6^A regulators that vary among cancer types, may be developed as an independent prognostic factor for acquired therapy resistance against immune checkpoint inhibitors.

Integrating multiple m^6^A regulators’ modification patterns to evaluate and identify different TME landscapes has revealed that m^6^A modification is important in shaping different TME landscapes. For instance, in gastric cancer, m^6^A modification patterns mediated by multiple m^6^A regulators are highly correlated with several immune-associated biological processes, thus leading to the apparent heterogeneity and complexity of immune cell infiltration characteristics in the immune microenvironment of gastric cancer^84^. Another study has demonstrated the correlation between m^6^A modification and the immune landscape in ccRCC and verified that the m^6^A score is an independent prognostic factor for PD-1 blockade therapy response in patients with advanced ccRCC^85^. Additionally, after systematic analysis of 21 m^6^A regulators in adrenocortical carcinoma samples from TCGA and GEO databases, Jin et al.^86^ have identified 3 m^6^A modification patterns with various clinical outcomes, demonstrating that the m^6^A clusters are strongly associated with immune infiltration in adrenocortical carcinoma. More recently, Xu et al.^87^ have confirmed a significant difference in m^6^A regulator levels between patients with high and low lung squamous cell carcinoma (LUSC). Compared with low-risk LUSC patients, high-risk LUSC patients have significantly lower rates of ALKBH5, METTL3, HNRNPC, and KIAA1429, and display more promising responses to anti-PD-L1 immunotherapy. Subsequently, through developing the five m^6^A regulators as a prognostic risk signature in lung adenocarcinoma, another study has similarly shown that patients with high-risk, rather than low-risk, lung adenocarcinoma have significantly higher PD-L1 levels, a lower proportion of CD8^+^ T cells, and better response to checkpoint blockade therapy, thus indicating that these five m^6^A regulators are highly correlated with immune infiltration levels and the immune checkpoint blockade response^88^. Similar studies have been performed in breast cancer and pancreatic ductal adenocarcinoma in addition to the above tumor types. He et al.^89^ have categorized 775 breast cancer patients into 2 subgroups and revealed that the high methylation group showed higher expression of tumor-infiltrating CD8^+^ T cells and activated NK cells, but less PD-L1, PD-L2, TIM3, and CCR4 than patients in the lower RNA methylation group. Moreover, tumors with high m^6^A scores are characterized by diminished immune infiltration and T cell exhaustion in patients with pancreatic ductal adenocarcinoma^90^. Briefly, other analyses in head and neck squamous cell carcinoma and esophageal cancer have linked the m^6^A score with the tumor immune microenvironment landscape, thus confirming that the m^6^A score is suitable for development as an immune therapeutic target and viable prognostic biomarker^91,92^.

Together, increasing evidence demonstrates that m^6^A modification patterns in a variety of cancer types have an indispensable role in overall TME infiltration characteristics and immune evasive phenotypes. A comprehensive evaluation of m^6^A modification patterns *via* their interactions with multiple m^6^A regulators in individual patients would not only enhance understanding of the tumor immune landscape but also provide novel insights regarding current immunotherapeutic strategies. Targeting m^6^A regulators or m^6^A phenotype-associated genes to restore the m^6^A modification level and reverse aberrant TME cell infiltration may provide effective anti-tumor therapeutics for patients with cancer.

## Conclusions and perspectives

In recent years, extensive epigenetic studies have gradually increased knowledge regarding the pivotal role of m^6^A methylation and its underlying molecular mechanisms in cancers. Currently, m^6^A modifications have been demonstrated to play crucial roles in metabolism, drug resistance, and metastasis in a variety of malignant tumors. Extensive research efforts focusing on m^6^A regulators have confirmed that targeting dysregulated m^6^A regulators is an attractive strategy for cancer therapy. In recent years, specific small molecule inhibitors of m^6^A modification have gradually been developed.

In the past few years, small molecule inhibitors targeting FTO, such as the natural product rhein^93^ and meclofenamic acid^94^, have been identified and used to regulate aberrant m^6^A levels. However, more effective and selective inhibitors have been discovered, including FB23, FB23-2^95^, CS1, CS2^83^, and FTO-04^96^. In addition, one study has identified 2 inhibitors targeting ALKBH5, 2-[(1-hydroxy-2-oxo-2-phenylethyl)sulfanyl]acetic acid and 4-{[(furan-2-yl)methyl]amino}-1,2-diazinane-3,6-dione, through high-throughput virtual screening. The efficacy of these compounds has been validated on the basis of suppressed proliferation observed in 3 leukemia cell lines^97^. Recently, Yankova et al.^98^ have found that STM2457, an effective and selective inhibitor of METTL3, displays promising anti-leukemia effects. Moreover, STM2457 exerts effects in a PDX model and a primary mouse model. In summary, targeting multiple m^6^A regulators should contribute to establishing more effective treatments for cancer patients and provide new insights into RNA m^6^A modification in cancer.

Our review is limited by the insufficient studies providing details of the direct connection between the TME and m^6^A methylation. The molecular mechanisms underlying how these m^6^A regulators interact with one another in a variety of cancers, and how they regulate the anti-tumor immune response, must be further explored. First, the establishment of more stable and sensitive sequencing technology will be essential. To date, m^6^A-sequencing technology cannot reach the single-cell level. However, an RNA methylation omics map at the single-cell level will be needed to uncover the detailed interplay between RNA epigenetics and tumor immunology. In the future, an m^6^A modification omics map at the single-cell level of immune cells in tumor tissues, adjacent tissues and lymph nodes should enable additional advances.

**Figure 2:**
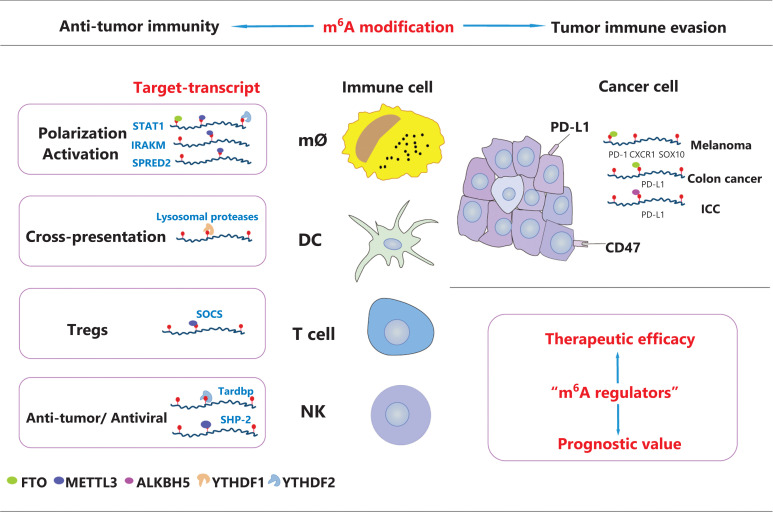
Key regulation of m^6^A modification in tumor immunity. As shown, the m^6^A modification is involved in anti-tumor immunity and tumor immune evasion. m^6^A methylation plays an essential role in the maintenance of immune cell homeostasis and function. Moreover, m^6^A modification patterns have an indispensable role in overall TME infiltration and the immune evasive phenotype. The close correlation between m^6^A-related risk signatures and the tumor immune microenvironment landscape supports the prognostic value and therapeutic efficacy of m^6^A regulators in a variety of cancer types.

In summary, m^6^A methylation is closely associated with immunological suppression and tumor progression **(Figure 2)**. A better understanding of the crosstalk between m^6^A modification and tumor immunoregulation is important to reveal new pathogenic pathways and develop promising therapeutic targets for cancers.

## References

[1] Sigalotti L, Covre A, Fratta E, Parisi G, Colizzi F, Rizzo A (2010). Epigenetics of human cutaneous melanoma: setting the stage for new therapeutic strategies. J Transl Med.

[2] Dunn J, Rao S (2017). Epigenetics and immunotherapy: the current state of play. Mol Immunol.

[3] Desrosiers R, Friderici K, Rottman F (1974). Identification of methylated nucleosides in messenger RNA from Novikoff hepatoma cells. Proc Natl Acad Sci U S A.

[4] Dominissini D, Moshitch-Moshkovitz S, Schwartz S, Salmon-Divon M, Ungar L, Osenberg S (2012). Topology of the human and mouse m6A RNA methylomes revealed by m6A-seq. Nature.

[5] Meyer KD, Saletore Y, Zumbo P, Elemento O, Mason CE, Jaffrey SR (2012). Comprehensive analysis of mRNA methylation reveals enrichment in 3’ UTRs and near stop codons. Cell.

[6] Zhang C, Chen Y, Sun B, Wang L, Yang Y, Ma D (2017). m(6)A modulates haematopoietic stem and progenitor cell specification. Nature.

[7] Fustin JM, Doi M, Yamaguchi Y, Hida H, Nishimura S, Yoshida M (2013). RNA-methylation-dependent RNA processing controls the speed of the circadian clock. Cell.

[8] Xiang Y, Laurent B, Hsu CH, Nachtergaele S, Lu Z, Sheng W (2017). RNA m(6)A methylation regulates the ultraviolet-induced DNA damage response. Nature.

[9] Lence T, Akhtar J, Bayer M, Schmid K, Spindler L, Ho CH (2016). m(6)A modulates neuronal functions and sex determination in Drosophila. Nature.

[10] Boccaletto P, Machnicka MA, Purta E, Piatkowski P, Baginski B, Wirecki TK (2018). MODOMICS: a database of RNA modification pathways. 2017 update. Nucleic Acids Res.

[11] Batista PJ (2017). The RNA modification N(6)-methyladenosine and its implications in human disease. Genom Proteom Bioinf.

[12] Wang X, Feng J, Xue Y, Guan Z, Zhang D, Liu Z (2017). Corrigendum: structural basis of N(6)-adenosine methylation by the METTL3-METTL14 complex. Nature.

[13] Liu J, Yue Y, Han D, Wang X, Fu Y, Zhang L (2014). A METTL3-METTL14 complex mediates mammalian nuclear RNA N6-adenosine methylation. Nat Chem Biol.

[14] Lin S, Choe J, Du P, Triboulet R, Gregory RI (2016). The m(6)A methyltransferase METTL3 promotes translation in human cancer cells. Mol Cell.

[15] Choe J, Lin S, Zhang W, Liu Q, Wang L, Ramirez-Moya J (2018). mRNA circularization by METTL3-eIF3h enhances translation and promotes oncogenesis. Nature.

[16] Ping XL, Sun BF, Wang L, Xiao W, Yang X, Wang WJ (2014). Mammalian WTAP is a regulatory subunit of the RNA N6-methyladenosine methyltransferase. Cell Res.

[17] Yue Y, Liu J, Cui X, Cao J, Luo G, Zhang Z (2018). VIRMA mediates preferential m(6)A mRNA methylation in 3’UTR and near stop codon and associates with alternative polyadenylation. Cell Discov.

[18] Shima H, Matsumoto M, Ishigami Y, Ebina M, Muto A, Sato Y (2017). S-Adenosylmethionine synthesis is regulated by selective N(6)-Adenosine methylation and mRNA degradation involving METTL16 and YTHDC1. Cell Rep.

[19] Warda AS, Kretschmer J, Hackert P, Lenz C, Urlaub H, Hobartner C (2017). Human METTL16 is a N(6)-methyladenosine (m(6)A) methyltransferase that targets pre-mRNAs and various non-coding RNAs. EMBO Rep.

[20] Jia G, Fu Y, Zhao X, Dai Q, Zheng G, Yang Y (2011). N6-methyladenosine in nuclear RNA is a major substrate of the obesity-associated FTO. Nat Chem Biol.

[21] Tang C, Klukovich R, Peng H, Wang Z, Yu T, Zhang Y (2018). ALKBH5-dependent m6A demethylation controls splicing and stability of long 3’-UTR mRNAs in male germ cells. Proc Natl Acad Sci U S A.

[22] Wang X, Lu Z, Gomez A, Hon GC, Yue Y, Han D (2014). N6-methyladenosine-dependent regulation of messenger RNA stability. Nature.

[23] Hsu PJ, Zhu Y, Ma H, Guo Y, Shi X, Liu Y (2017). Ythdc2 is an N(6)-methyladenosine binding protein that regulates mammalian spermatogenesis. Cell Res.

[24] Wang X, Zhao BS, Roundtree IA, Lu Z, Han D, Ma H (2015). N(6)-methyladenosine modulates messenger RNA translation efficiency. Cell.

[25] Shi H, Wang X, Lu Z, Zhao BS, Ma H, Hsu PJ (2017). YTHDF3 facilitates translation and decay of N(6)-methyladenosine-modified RNA. Cell Res.

[26] Roundtree IA, Luo GZ, Zhang Z, Wang X, Zhou T, Cui Y (2017). YTHDC1 mediates nuclear export of N(6)-methyladenosine methylated mRNAs. eLife.

[27] Anita R, Paramasivam A, Priyadharsini JV, Chitra S (2020). The m6A readers YTHDF1 and YTHDF3 aberrations associated with metastasis and predict poor prognosis in breast cancer patients. Am J Cancer Res.

[28] Li A, Chen YS, Ping XL, Yang X, Xiao W, Yang Y (2017). Cytoplasmic m(6)A reader YTHDF3 promotes mRNA translation. Cell Res.

[29] Wojtas MN, Pandey RR, Mendel M, Homolka D, Sachidanandam R, Pillai RS (2017). Regulation of m(6)A transcripts by the 3’-->5’ RNA helicase YTHDC2 is essential for a successful meiotic program in the mammalian germline. Mol Cell.

[30] Xiao W, Adhikari S, Dahal U, Chen YS, Hao YJ, Sun BF (2016). Nuclear m(6)A Reader YTHDC1 Regulates mRNA Splicing. Mol Cell.

[31] Huang H, Weng H, Sun W, Qin X, Shi H, Wu H (2018). Recognition of RNA N(6)-methyladenosine by IGF2BP proteins enhances mRNA stability and translation. Nat Cell Biol.

[32] Alarcon CR, Goodarzi H, Lee H, Liu X, Tavazoie S, Tavazoie SF (2015). HNRNPA2B1 is a mediator of m(6)A-dependent nuclear RNA processing events. Cell.

[33] Zheng Q, Hou J, Zhou Y, Li Z, Cao X (2017). The RNA helicase DDX46 inhibits innate immunity by entrapping m(6)A-demethylated antiviral transcripts in the nucleus. Nat Immunol.

[34] Kariko K, Buckstein M, Ni H, Weissman D (2005). Suppression of RNA recognition by Toll-like receptors: the impact of nucleoside modification and the evolutionary origin of RNA. Immunity.

[35] Chen YG, Chen R, Ahmad S, Verma R, Kasturi SP, Amaya L (2019). N6-methyladenosine modification controls circular RNA immunity. Mol Cell.

[36] Sica A, Erreni M, Allavena P, Porta C (2015). Macrophage polarization in pathology. Cell Mol Life Sci.

[37] Shrivastava R, Asif M, Singh V, Dubey P, Ahmad Malik S, Lone MU (2019). M2 polarization of macrophages by Oncostatin M in hypoxic tumor microenvironment is mediated by mTORC2 and promotes tumor growth and metastasis. Cytokine.

[38] Colegio OR, Chu NQ, Szabo AL, Chu T, Rhebergen AM, Jairam V (2014). Functional polarization of tumour-associated macrophages by tumour-derived lactic acid. Nature.

[39] Murray PJ, Allen JE, Biswas SK, Fisher EA, Gilroy DW, Goerdt S (2014). Macrophage activation and polarization: nomenclature and experimental guidelines. Immunity.

[40] Liu Y, Liu Z, Tang H, Shen Y, Gong Z, Xie N (2019). The N(6)-methyladenosine (m(6)A)-forming enzyme METTL3 facilitates M1 macrophage polarization through the methylation of *STAT1* mRNA. Am J Physiol Cell Physiol.

[41] Tong J, Wang X, Liu Y, Ren X, Wang A, Chen Z (2021). Pooled CRISPR screening identifies m(6)A as a positive regulator of macrophage activation. Sci Adv.

[42] Yin H, Zhang X, Yang P, Zhang X, Peng Y, Li D (2021). RNA m6A methylation orchestrates cancer growth and metastasis via macrophage reprogramming. Nat Commun.

[43] Dong L, Chen C, Zhang Y, Guo P, Wang Z, Li J (2021). The loss of RNA N(6)-adenosine methyltransferase Mettl14 in tumor-associated macrophages promotes CD8(+) T cell dysfunction and tumor growth. Cancer Cell.

[44] Merad M, Salmon H (2015). Cancer: a dendritic-cell brake on antitumour immunity. Nature.

[45] Han D, Liu J, Chen C, Dong L, Liu Y, Chang R (2019). Anti-tumour immunity controlled through mRNA m(6)A methylation and YTHDF1 in dendritic cells. Nature.

[46] Li HB, Tong J, Zhu S, Batista PJ, Duffy EE, Zhao J (2017). m(6)A mRNA methylation controls T cell homeostasis by targeting the IL-7/STAT5/SOCS pathways. Nature.

[47] Tong J, Cao G, Zhang T, Sefik E, Amezcua Vesely MC, Broughton JP (2018). m(6)A mRNA methylation sustains Treg suppressive functions. Cell Res.

[48] Zheng Z, Zhang L, Cui X-L, Yu X, Hsu PJ, Lyu R (2020). Control of Early B cell development by the RNA N-Methyladenosine methylation. Cell Rep.

[49] Sun JC, Lanier LL (2011). NK cell development, homeostasis and function: parallels with CD8(+) T cells. Nat Rev Immunol.

[50] Fauriat C, Long EO, Ljunggren HG, Bryceson YT (2010). Regulation of human NK-cell cytokine and chemokine production by target cell recognition. Blood.

[51] Ma S, Yan J, Barr T, Zhang J, Chen Z, Wang LS (2021). The RNA m6A reader YTHDF2 controls NK cell antitumor and antiviral immunity. J Exp Med.

[52] Song H, Song J, Cheng M, Zheng M, Wang T, Tian S (2021). METTL3-mediated m(6)A RNA methylation promotes the anti-tumour immunity of natural killer cells. Nat Commun.

[53] Beatty GL, Gladney WL (2015). Immune escape mechanisms as a guide for cancer immunotherapy. Clin Cancer Res.

[54] Rizvi NA, Hellmann MD, Snyder A, Kvistborg P, Makarov V, Havel JJ (2015). Cancer immunology. Mutational landscape determines sensitivity to PD-1 blockade in non-small cell lung cancer. Science.

[55] Cristescu R, Mogg R, Ayers M, Albright A, Murphy E, Yearley J (2018). Pan-tumor genomic biomarkers for PD-1 checkpoint blockade-based immunotherapy. Science.

[56] Wang L, Hui H, Agrawal K, Kang Y, Li N, Tang R (2020). m(6) A RNA methyltransferases METTL3/14 regulate immune responses to anti-PD-1 therapy. EMBO J.

[57] Yang S, Wei J, Cui YH, Park G, Shah P, Deng Y (2019). m(6)A mRNA demethylase FTO regulates melanoma tumorigenicity and response to anti-PD-1 blockade. Nat Commun.

[58] Tsuruta N, Tsuchihashi K, Ohmura H, Yamaguchi K, Ito M, Ariyama H (2020). RNA N6-methyladenosine demethylase FTO regulates PD-L1 expression in colon cancer cells. Biochem Biophys Res Commun.

[59] Qiu X, Yang S, Wang S, Wu J, Zheng B, Wang K (2021). M(6)A demethylase ALKBH5 regulates PD-L1 expression and tumor immunoenvironment in intrahepatic cholangiocarcinoma. Cancer Res.

[60] Zhao H, Wang J, Kong X, Li E, Liu Y, Du X (2016). CD47 promotes tumor invasion and metastasis in non-small cell lung cancer. Sci Rep.

[61] Fan Z, Gao Y, Zhang W, Yang G, Liu P, Xu L (2021). METTL3/IGF2BP1/CD47 contributes to the sublethal heat treatment induced mesenchymal transition in HCC. Biochem Biophys Res Commun.

[62] Guo X, Zhao Y, Yan H, Yang Y, Shen S, Dai X (2017). Single tumor-initiating cells evade immune clearance by recruiting type II macrophages. Genes Dev.

[63] Wang G, Lu X, Dey P, Deng P, Wu CC, Jiang S (2016). Targeting YAP-dependent MDSC infiltration impairs tumor progression. Cancer Discov.

[64] Murakami S, Shahbazian D, Surana R, Zhang W, Chen H, Graham GT (2017). Yes-associated protein mediates immune reprogramming in pancreatic ductal adenocarcinoma. Oncogene.

[65] Suh JH, Won KY, Kim GY, Bae GE, Lim SJ, Sung JY (2015). Expression of tumoral FOXP3 in gastric adenocarcinoma is associated with favorable clinicopathological variables and related with Hippo pathway. Int J Clin Exp Pathol.

[66] Chen J, Sun Y, Xu X, Wang D, He J, Zhou H (2017). YTH domain family 2 orchestrates epithelial-mesenchymal transition/proliferation dichotomy in pancreatic cancer cells. Cell Cycle (Georgetown, Tex.).

[67] Ni W, Yao S, Zhou Y, Liu Y, Huang P, Zhou A (2019). Long noncoding RNA GAS5 inhibits progression of colorectal cancer by interacting with and triggering YAP phosphorylation and degradation and is negatively regulated by the m(6)A reader YTHDF3. Mol Cancer.

[68] Zhang X, Xu Y, Qian Z, Zheng W, Wu Q, Chen Y (2018). circRNA_104075 stimulates YAP-dependent tumorigenesis through the regulation of HNF4a and may serve as a diagnostic marker in hepatocellular carcinoma. Cell Death Dis.

[69] Jin D, Guo J, Wu Y, Du J, Yang L, Wang X (2019). m(6)A mRNA methylation initiated by METTL3 directly promotes YAP translation and increases YAP activity by regulating the MALAT1-miR-1914-3p-YAP axis to induce NSCLC drug resistance and metastasis. J Hematol Oncol.

[70] Jin D, Guo J, Wu Y, Yang L, Wang X, Du J (2020). m(6)A demethylase ALKBH5 inhibits tumor growth and metastasis by reducing YTHDFs-mediated YAP expression and inhibiting miR-107/LATS2-mediated YAP activity in NSCLC. Mol Cancer.

[71] Sweis RF, Spranger S, Bao R, Paner GP, Stadler WM, Steinberg G (2016). Molecular drivers of the Non-T-cell-inflamed tumor microenvironment in urothelial bladder cancer. Cancer Immunol Res.

[72] Seiwert TY, Zuo Z, Keck MK, Khattri A, Pedamallu CS, Stricker T (2015). Integrative and comparative genomic analysis of HPV-positive and HPV-negative head and neck squamous cell carcinomas. Clin Cancer Res.

[73] Jimenez-Sanchez A, Memon D, Pourpe S, Veeraraghavan H, Li Y, Vargas HA (2017). Heterogeneous tumor-immune microenvironments among differentially growing metastases in an ovarian cancer patient. Cell.

[74] Mlecnik B, Bindea G, Angell HK, Maby P, Angelova M, Tougeron D (2016). Integrative analyses of colorectal cancer show immunoscore is a stronger predictor of patient survival than microsatellite instability. Immunity.

[75] Spranger S, Gajewski TF (2018). Impact of oncogenic pathways on evasion of antitumour immune responses. Nat Rev Cancer.

[76] Martin-Orozco E, Sanchez-Fernandez A, Ortiz-Parra I, Ayala-San Nicolas M (2019). WNT signaling in tumors: the way to evade drugs and immunity. Front Immunol.

[77] Bai Y, Yang C, Wu R, Huang L, Song S, Li W (2019). YTHDF1 regulates tumorigenicity and cancer stem cell-like activity in human colorectal carcinoma. Front Oncol.

[78] Liu L, Wang J, Sun G, Wu Q, Ma J, Zhang X (2019). m(6)A mRNA methylation regulates CTNNB1 to promote the proliferation of hepatoblastoma. Mol Cancer.

[79] Miao W, Chen J, Jia L, Ma J, Song D (2019). The m6A methyltransferase METTL3 promotes osteosarcoma progression by regulating the m6A level of LEF1. Biochem Biophys Res Commun.

[80] Liu X, Qin J, Gao T, Li C, He B, Pan B (2020). YTHDF1 facilitates the progression of hepatocellular carcinoma by promoting FZD5 mRNA translation in an m6A-dependent manner. Mol Ther Nucleic Acids.

[81] Shriwas O, Mohapatra P, Mohanty S, Dash R (2020). The impact of m6A RNA modification in therapy resistance of cancer: implication in chemotherapy, radiotherapy, and immunotherapy. Front Oncol.

[82] Li N, Kang Y, Wang L, Huff S, Tang R, Hui H (2020). ALKBH5 regulates anti-PD-1 therapy response by modulating lactate and suppressive immune cell accumulation in tumor microenvironment. Proc Natl Acad Sci U S A.

[83] Su R, Dong L, Li Y, Gao M, Han L, Wunderlich M (2020). Targeting FTO suppresses cancer stem cell maintenance and immune evasion. Cancer Cell.

[84] Zhang B, Wu Q, Li B, Wang D, Wang L, Zhou YL (2020). m(6)A regulator-mediated methylation modification patterns and tumor microenvironment infiltration characterization in gastric cancer. Mol Cancer.

[85] Zhong J, Liu Z, Cai C, Duan X, Deng T, Zeng G (2021). m(6)A modification patterns and tumor immune landscape in clear cell renal carcinoma. J Immunother Cancer.

[86] Jin Y, Wang Z, He D, Zhu Y, Hu X, Gong L (2021). Analysis of m6A-Related signatures in the tumor immune microenvironment and identification of clinical prognostic regulators in adrenocortical carcinoma. Front Immunol.

[87] Xu F, Zhang H, Chen J, Lin L, Chen Y (2020). Immune signature of T follicular helper cells predicts clinical prognostic and therapeutic impact in lung squamous cell carcinoma. Int Immunopharmacol.

[88] Wu X, Sheng H, Wang L, Xia P, Wang Y, Yu L (2021). A five-m6A regulatory gene signature is a prognostic biomarker in lung adenocarcinoma patients. Aging (Albany NY).

[89] He X, Tan L, Ni J, Shen G (2021). Expression pattern of m(6)A regulators is significantly correlated with malignancy and antitumor immune response of breast cancer. Cancer Gene Ther.

[90] Zhou Z, Zhang J, Xu C, Yang J, Zhang Y, Liu M (2021). An integrated model of N6-methyladenosine regulators to predict tumor aggressiveness and immune evasion in pancreatic cancer. EBioMedicine.

[91] Zhao H, Xu Y, Xie Y, Zhang L, Gao M, Li S (2021). m6A regulators is differently expressed and correlated with immune response of Esophageal cancer. Front Cell Dev Biol.

[92] Yi L, Wu G, Guo L, Zou X, Huang P (2020). Comprehensive analysis of the PD-L1 and immune infiltrates of m(6)A RNA methylation regulators in head and neck squamous cell carcinoma. Mol Therapy Nucleic Acids.

[93] Chen B, Ye F, Yu L, Jia G, Huang X, Zhang X (2012). Development of cell-active N6-methyladenosine RNA demethylase FTO inhibitor. J Am Chem Soc.

[94] Huang Y, Yan J, Li Q, Li J, Gong S, Zhou H (2015). Meclofenamic acid selectively inhibits FTO demethylation of m6A over ALKBH5. Nucleic Acids Res.

[95] Huang Y, Su R, Sheng Y, Dong L, Dong Z, Xu H (2019). Small-molecule targeting of oncogenic FTO demethylase in acute myeloid leukemia. Cancer Cell.

[96] Huff S, Tiwari SK, Gonzalez GM, Wang Y, Rana TM (2021). m(6)A-RNA Demethylase FTO inhibitors impair self-renewal in glioblastoma stem cells. ACS Chem Biol.

[97] Selberg S, Seli N, Kankuri E, Karelson M (2021). Rational design of novel anticancer small-molecule RNA m6A demethylase ALKBH5 inhibitors. ACS Omega.

[98] Yankova E, Blackaby W, Albertella M, Rak J, De Braekeleer E, Tsagkogeorga G (2021). Small-molecule inhibition of METTL3 as a strategy against myeloid leukaemia. Nature.

